# Systematic Review and Meta-Analysis on Central and/or Peripheral Nervous System Demyelination in Patients Treated With Tumor Necrosis Factor Alpha (TNF-α) Inhibitors

**DOI:** 10.7759/cureus.116108

**Published:** 2026-09-11

**Authors:** Shamma Al Nokhatha, Robert Harrington, Hamda Kamalboor

**Affiliations:** 1 Rheumatology, Sheikh Tahnoon Bin Mohammed Medical City and Tawam Hospital, Al Ain, ARE; 2 Internal Medicine, College of Medicine and Health Sciences, Al Ain, ARE; 3 Rheumatology, St. James's Hospital, Dublin, IRL; 4 Neurology, Rashid Hospital, Dubai, ARE

**Keywords:** central nervous system, demyelination, immune-mediated inflammatory diseases, peripheral nervous system, tnf-alpha inhibitors

## Abstract

Tumor necrosis factor alpha inhibitors (TNFi) are widely used for autoimmune diseases, but concerns exist regarding their association with demyelinating disorders affecting the central nervous system (CNS) and peripheral nervous system (PNS). The objective is to systematically evaluate the association between TNF inhibitor exposure and the risk of CNS and PNS demyelination.

A systematic review and meta-analysis was conducted following PRISMA 2020 guidelines. PubMed, Scopus, Embase, and the Cochrane Library were searched for studies published between January 2000 and February 2026. Observational cohort and case-control studies reporting TNFi exposure and demyelinating outcomes were included. Risk of bias was assessed using the Risk Of Bias In Non-randomized Studies - of Interventions (ROBINS-I) tool and visualized using robvis (Luke A. McGuinness, Bristol, UK). Random-effects meta-analysis was performed using the metafor package (Wolfgang Viechtbauer, Maastricht University, Maastricht, Netherlands). Subgroup analyses were conducted for CNS and PNS outcomes. Publication bias was evaluated using funnel plots and Egger’s regression test.

Thirteen studies were included in the systematic review, with 11 eligible for meta-analysis. TNFi exposure was associated with an increased risk of demyelinating disorders affecting both CNS and PNS, with pooled random-effects estimates of RR 1.46 (95% CI 1.09-1.95) for CNS demyelination and RR 2.00 (95% CI 1.19-3.38) for PNS demyelination. Moderate heterogeneity was observed overall (I² = 54.2%), with subgroup heterogeneity of 50.4% for CNS and 47.4% for PNS outcomes. Subgroup analysis showed no significant difference between CNS and PNS risk (p = 0.334). Risk-of-bias assessment demonstrated predominantly low-to-moderate risk of bias, though three single-arm studies without a comparator group were judged to have serious risk of bias; Egger’s test showed no significant publication bias.

TNFi therapy is associated with increased risk of CNS and PNS demyelinating disorders. Clinicians should monitor neurological symptoms during treatment. Further prospective studies are needed to clarify mechanisms and identify high-risk populations.

## Introduction and background

Tumor necrosis factor alpha (TNF-α) is produced predominantly by the astrocytes, microglia, and neurons within the central nervous system (CNS) under pathological conditions. It functions as a proinflammatory cytokine, contributing to tissue inflammation and end-organ damage in several autoimmune diseases [1]. Anti-TNF-α medications inhibit the pro-inflammatory effects and reduce symptoms [2,3]. Immune-mediated inflammatory diseases (IMIDs), such as inflammatory bowel disease (IBD), rheumatoid arthritis (RA), and psoriatic arthritis (PsA), are linked to significant systemic inflammation and frequently necessitate the use of immunomodulatory treatments [4]. Whether used alone or in combination with other anti-inflammatory or disease-modifying antirheumatic drugs (DMARDs), TNF-α blockers are highly effective for treating chronic IMIDs such as ankylosing spondylitis (AS), RA, and IBD [3,5,6]. Terminologies such as TNF-α blockers, anti-TNF-α agents, TNF-α inhibitors (TNFi), and TNF-α antagonists are often used interchangeably [7]. Currently, five TNFi - infliximab, golimumab, adalimumab, certolizumab, and etanercept - are approved for clinical use [7]. However, concerns persist regarding their safety, especially their potential to induce demyelinating disorders, characterized by damage to the protective myelin sheath surrounding nerve fibers, in the CNS and peripheral nervous system (PNS) [8,9]. Several reports suggest an association between TNFi and CNS demyelinating disorders, such as multiple sclerosis (MS) and optic neuritis (ON), as well as PNS demyelinating disorders like Guillain-Barré syndrome (GBS) and chronic inflammatory demyelinating polyneuropathy (CIDP) [10,11]. However, it remains uncertain whether TNFi directly cause demyelination or merely trigger its onset in predisposed individuals [12,13]. One hypothesis suggests that TNFi could promote CNS demyelination and inflammation by blocking TNF-α signaling via TNF type-2 receptors. This action may increase autoimmunity due to the decreased survival of regulatory T cells or alter the cytokine balance [1]. Although the precise mechanism of TNFi-induced demyelination in the PNS remains unclear, similar mechanisms are thought to play a role in this process [14-16]. Despite their therapeutic benefits, the exact mechanisms underlying TNFi-induced demyelination remain unclear. Although TNFi-associated demyelinating events are uncommon, their potential clinical consequences are important. However, the magnitude of this risk and whether it differs between CNS and PNS manifestations remain uncertain. Therefore, this systematic review and meta-analysis aimed to synthesize the available evidence and evaluate and compare the risk of CNS and PNS demyelinating outcomes associated with TNFi exposure.

## Review

Materials and methods

Study Design and Reporting Guidelines

This systematic review and meta-analysis was conducted in accordance with the Preferred Reporting Items for Systematic Reviews and Meta-Analyses (PRISMA) 2020 guidelines to ensure transparent and reproducible reporting. The PRISMA 2020 flow diagram was generated using the PRISMA2020 R package (Neal R. Haddaway (Müncheberg, Germany), Matthew J. Page (Melbourne, Australia), Chris C. Pritchard (Nottingham, UK), and Luke A. McGuinness (Bristol, UK) and its accompanying Shiny web application (Posit, Boston, MA), which facilitates accurate visualization of the study selection process and enhances reporting transparency [17]. Risk-of-bias assessments were visualized using the robvis R package (Luke A. McGuinness, Bristol, UK) and Shiny web application, which provides standardized graphical summaries of risk-of-bias domains for non-randomized studies [18]. The study protocol was developed prior to data extraction and analysis to ensure methodological rigor and consistency. The study was registered in PROSPERO with the registration number CRD42025621062.

Literature Search Strategy

A comprehensive literature search was performed to identify studies evaluating the association between TNFi exposure and the risk of CNS or PNS demyelinating disorders. Electronic databases including PubMed, Scopus, Embase, and the Cochrane Library were systematically searched for studies published between January 2000 and February 2026. The search strategy incorporated a combination of controlled vocabulary and free-text keywords related to TNF inhibitors and demyelinating outcomes, including terms such as “tumor necrosis factor inhibitors,” “TNF inhibitors,” “biologic therapy,” “demyelination,” “multiple sclerosis,” “optic neuritis,” “Guillain-Barré syndrome,” and “inflammatory neuropathy.” Boolean operators and database-specific filters were applied to optimize sensitivity and specificity. In addition, reference lists of eligible articles were manually reviewed to identify any additional relevant studies not captured in the initial database search. The complete database-specific search strings, including MeSH/Emtree terms, Boolean logic, and applied filters for all four databases, are provided in Appendix 1. Grey literature sources, trial registries, and pharmacovigilance databases (e.g., FDA Adverse Event Reporting System (FAERS), EudraVigilance, ClinicalTrials.gov) were not searched, which is acknowledged as a limitation.

Study Selection

All identified records were independently screened by SAA and RH to determine eligibility. Titles and abstracts were initially reviewed to exclude clearly irrelevant studies, followed by full-text assessment of potentially eligible articles. Eligibility decisions were verified by all authors to ensure accuracy and consensus. Studies were included if they employed observational cohort or case-control designs, evaluated TNF inhibitor exposure, reported CNS or PNS demyelinating outcomes, included an appropriate comparator group, and provided effect estimates such as risk ratios (RRs), odds ratios (ORs), hazard ratios (HRs), incidence rate ratios (IRRs), or sufficient data for calculation. Studies were excluded if they were review articles, editorials, case reports, animal studies, mechanistic investigations, lacked comparator groups, or did not report demyelinating outcomes or sufficient data for risk estimation. The complete study selection process is illustrated in the PRISMA 2020 flow diagram. A total of 13 studies met the eligibility criteria and were included in the systematic review, of which 11 provided sufficient quantitative data for inclusion in the meta-analysis.

Data Extraction

Data extraction was performed independently using a standardized extraction framework to ensure consistency and completeness. Extracted information included study characteristics such as first author, publication year, country, registry source, study design, sample size, and population characteristics. Details of TNF inhibitor exposure, comparator groups, and follow-up duration were also recorded. Outcome data included the type of demyelinating disorder (CNS or PNS), diagnostic criteria, and reported effect estimates with corresponding 95% confidence intervals. When studies reported multiple independent outcomes, such as both CNS and PNS demyelination, each outcome was treated as a separate analytical entry in subgroup analyses. All extracted data were cross-checked for accuracy prior to statistical analysis.

Risk of Bias Assessment

Risk of bias was evaluated using the Risk Of Bias In Non-randomized Studies - of Interventions (ROBINS-I) tool, which is specifically designed for assessing bias in observational studies of interventions. This tool assesses bias across seven domains, including bias due to confounding, participant selection, classification of interventions, deviations from intended interventions, missing data, outcome measurement, and selective reporting. Risk-of-bias assessments were performed independently by SAA and RH, with verification by all authors to ensure accuracy and consistency. Visual summaries of risk-of-bias assessments were generated using the robvis Shiny web application.

Statistical Analysis

Meta-analysis was conducted using RStudio (R version 4.5.2, R Foundation for Statistical Computing, Vienna, Austria)with the metafor package (Wolfgang Viechtbauer, Maastricht University, Maastricht, Netherlands). Reported effect estimates were harmonized by converting all risk estimates to the logarithmic scale (logRR), and corresponding variances were calculated from reported confidence intervals. Because the included studies reported five different effect-measure types (RR in three studies, OR in one study, HR in four studies, IRR in two studies, and standardized incidence ratio (SIR) in one study; full listing in Appendix 2), each non-RR measure was treated as approximating RR under the rare-outcome assumption, whereby OR, HR, and IRR converge toward RR as outcome incidence approaches zero; this assumption is reasonable given the low absolute incidence of demyelinating events reported across all included cohorts. A sensitivity analysis restricted to the RR-reporting studies, and pooled estimates stratified by measure type, are reported in the Sensitivity Analysis section and Appendix 2. Random-effects meta-analysis using the restricted maximum likelihood (REML) estimator was performed to account for between-study variability and heterogeneity. Heterogeneity among studies was assessed using Cochran’s Q test, the I² statistic, and tau² estimates. Publication bias was evaluated using funnel plots and Egger’s regression test. Sensitivity analyses were performed using leave-one-out procedures to assess the influence of individual studies on pooled estimates. Forest plots and funnel plots were generated using the metafor package to visually summarize pooled effect sizes and assess publication bias.

Subgroup Analysis

Subgroup analyses were conducted to evaluate differences in demyelination risk between CNS and PNS outcomes. Studies reporting CNS and PNS outcomes were categorized accordingly, and pooled effect estimates were calculated separately for each subgroup. Differences between subgroups were assessed using mixed-effects meta-regression models. This approach allowed evaluation of whether TNF inhibitor-associated demyelination risk differed significantly between CNS and PNS outcomes while accounting for between-study heterogeneity.

Results

Study Selection

The database search identified a total of 2,451 records, including 902 from Scopus, 107 from PubMed, 1,421 from Embase, and 21 from the Cochrane Library. After removal of duplicate records (n = 957) and records excluded for other reasons (n = 761), 733 records remained for title and abstract screening. Of these, 454 records were excluded based on relevance. Full-text articles were sought for 279 records, of which 24 reports could not be retrieved. A total of 255 full-text articles were assessed for eligibility. Among these, 242 studies were excluded due to lack of comparator groups (n = 71), animal study design (n = 124), or irrelevant outcomes (n = 47). Ultimately, 13 studies met the inclusion criteria and were included in the systematic review. Of these, 11 studies provided sufficient quantitative data and were included in the meta-analysis. The complete study selection process is illustrated in the PRISMA flow diagram (Figure 1).

**Figure 1:**
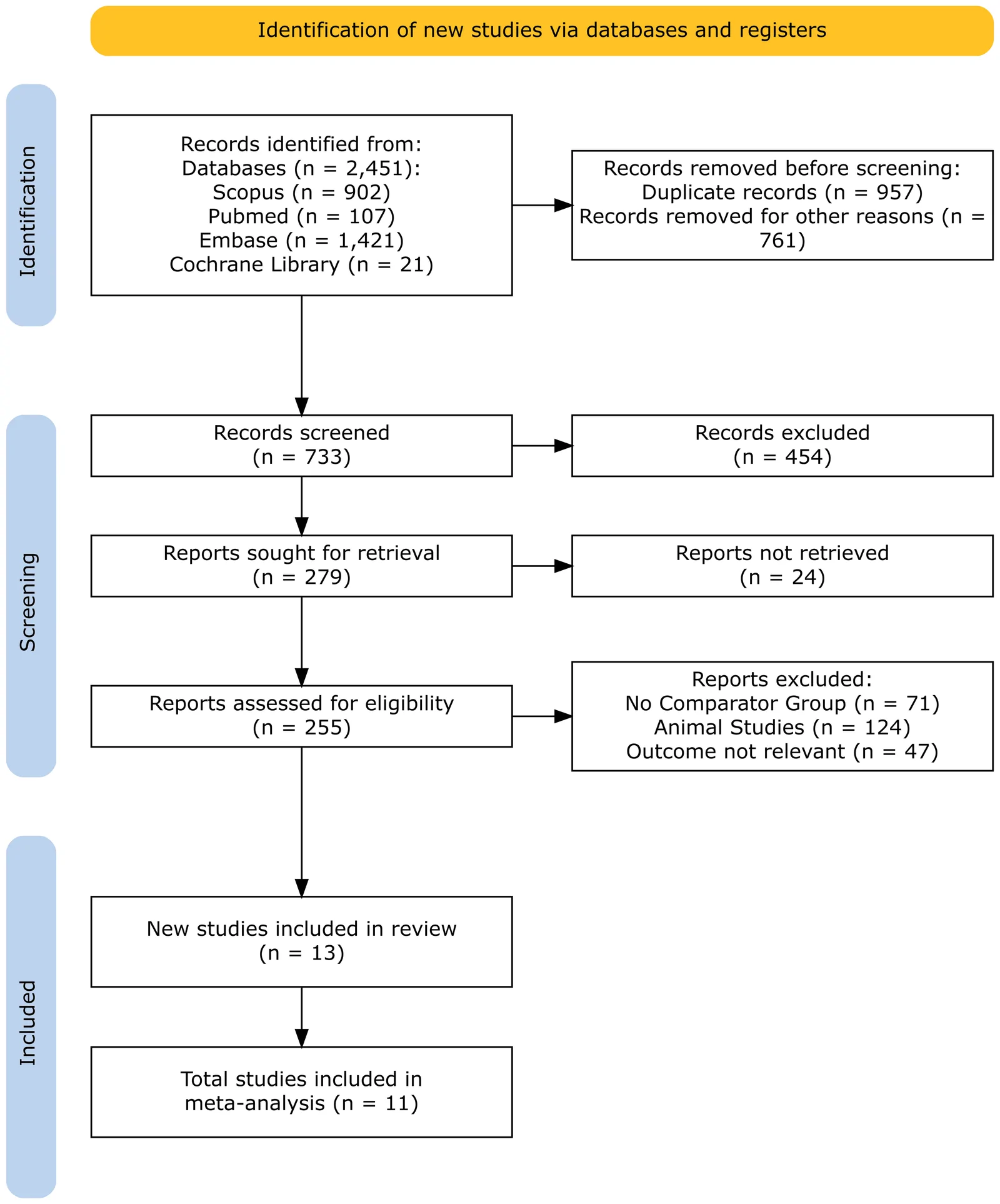
PRISMA 2020 flow diagram of study selection.

Study Characteristics

The 13 included studies were published between 2010 and 2023 and comprised observational cohort and case-control designs conducted across North America and Europe [7,19-30]. These studies evaluated TNF inhibitor exposure in patients with autoimmune diseases, including RA, IBD, AS, and PsA. Sample sizes ranged from fewer than 100 participants to more than 175,000 patients in large registry-based cohort studies. Nine studies evaluated CNS demyelinating outcomes, including MS, ON, and inflammatory demyelinating disorders, while four studies evaluated PNS demyelinating outcomes, including inflammatory polyneuropathy and peripheral neuropathy. Effect estimates were reported as HRs, RRs, ORs, or IRRs with corresponding confidence intervals. Eleven studies provided sufficient data for inclusion in the quantitative meta-analysis (Table 1) [30,31].

**Table 1:** Details of included studies (n = 13) AS, ankylosing spondylitis; BSRBR-RA, British Society for Rheumatology Biologics Register for Rheumatoid Arthritis; CNS, central nervous system; IBD, inflammatory bowel disease; PNS, peripheral nervous system; PsA, psoriatic arthritis; RA, rheumatoid arthritis; TNFi, tumor necrosis factor alpha inhibitor

Author	Year	Country/registry	Study design	Population	Sample size	Outcome type	Outcome category
Kunchok et al. [19]	2020	USA (Mayo Clinic)	Nested case-control	Autoimmune disease patients	212 cases/212 controls	CNS demyelination	CNS
Li et al. [21]	2023	Canada (Population registry)	Nested case-control	RA, IBD, PsA	314 cases/3140 controls	Multiple sclerosis	CNS
Delcoigne et al. [25]	2023	Nordic registries (Denmark, Sweden, Norway, Finland, Iceland)	Cohort	Rheumatoid arthritis, PsA, AS	33,883 TNFi users	CNS demyelination	CNS
Taylor et al. [20]	2021	UK (BSRBR-RA registry)	Prospective cohort	Rheumatoid arthritis	13,489 TNFi users	Demyelinating disease	CNS
De Felice et al. [22]	2015	USA	Retrospective cohort	Inflammatory bowel disease	9,095 patients	CNS demyelination	CNS
Avasarala et al. [28]	2021	USA	Retrospective cohort	Crohn’s disease	5,218 patients	Multiple sclerosis	CNS
Bernatsky et al. [30]	2010	Canada	Nested case-control	Rheumatoid arthritis	23,733 patients	CNS demyelination	CNS
Kopp et al. [26]	2020	Denmark and Sweden registries	Cohort	RA, PsA, AS	175,520 patients	Inflammatory polyneuropathy	PNS
Etminan et al. [27]	2019	USA insurance database	Nested case-control	Rheumatoid arthritis	61,570 patients	Peripheral neuropathy	PNS
Tsouni et al. [23]	2015	Switzerland	Cohort	Autoimmune disease patients	2,017 patients	Peripheral neuropathy	PNS
Ekinci et al. [24]	2021	Turkey	Case-control	Autoimmune disease patients	35 cases/37 controls	Peripheral neuropathy	PNS
Lucchini et al. [7]	2021	Italy	Case series	Autoimmune disease patients	10 patients	CNS demyelination	CNS
Kalén et al. [31]	2022	Sweden	Retrospective observational study	Neurological complications associated with TNFi	48 patients	clinically isolated syndrome, multiple sclerosis	CNS

Risk of Bias Assessment

Risk of bias was assessed using the ROBINS-I tool, and visual summaries were generated using the robvis Shiny web application (Figures 2A-2B). Most studies demonstrated low risk of bias in domains related to classification of interventions, deviations from intended interventions, missing data, and outcome measurement. Moderate risk of bias was observed in the domain of confounding across all studies, reflecting the inherent limitations of observational study designs. Moderate risk of bias was also identified in the selection of reported results for a small number of studies. Overall, most studies with a concurrent unexposed comparator group demonstrated low to moderate risk of bias. Three studies [7,23,31] were case series or referral-based cohorts without an unexposed comparator group and were judged to have serious overall risk of bias; their contribution to the review was therefore interpreted with caution (Appendix 3).

**Figure 2:**
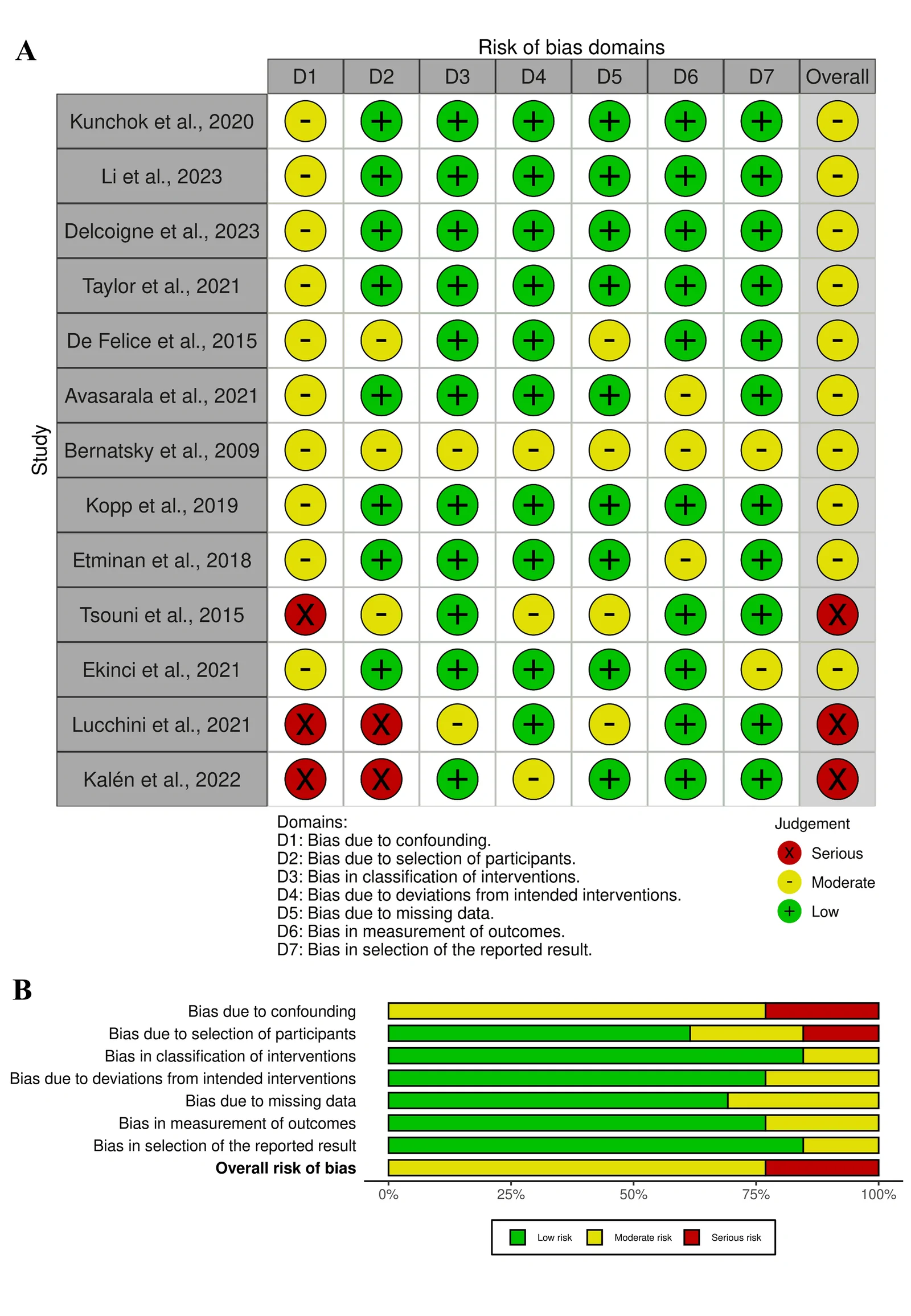
Risk-of-bias assessment of included studies. (A) Traffic light plot illustrating risk-of-bias judgments for individual studies across all Risk Of Bias In Non-randomized Studies - of Interventions (ROBINS-I) domains [7,19-30]. (B) Summary bar chart showing the proportion of studies classified as low, moderate, or serious risk of bias across all seven ROBINS-I domains.

Overall Meta-Analysis

A total of 11 studies were included in the quantitative meta-analysis evaluating the association between TNF inhibitor exposure and demyelinating disorders. Random-effects meta-analysis demonstrated moderate heterogeneity across studies (I² = 54.2%), indicating variability in effect sizes between studies. This heterogeneity likely reflects differences in study populations, underlying autoimmune diseases, TNF inhibitor exposure characteristics, and study methodologies. The pooled random-effects estimate across all 11 studies demonstrated an increased risk of demyelinating events associated with TNF inhibitor exposure (pooled RR 1.59, 95% CI 1.22-2.08; tau² = 0.0995; H² = 2.18; Q = 21.85, df = 10, p = 0.0159; 95% prediction interval 0.73-3.46).

CNS Demyelination Subgroup Analysis

Eight studies evaluating CNS demyelinating outcomes were included in the CNS subgroup meta-analysis. Random-effects meta-analysis demonstrated moderate heterogeneity (tau² = 0.0807; I² = 50.4%; H² = 2.01). The heterogeneity test was statistically significant (Q = 14.10, df = 7, p = 0.0494), indicating variability in CNS demyelination risk estimates across studies. Despite this heterogeneity, the pooled analysis demonstrated an increased risk of CNS demyelinating events associated with TNF inhibitor exposure (pooled RR 1.46, 95% CI 1.09-1.95), although the magnitude of risk varied across studies. These findings are illustrated in the subgroup forest plot (Figures 3A-3B).

**Figure 3:**
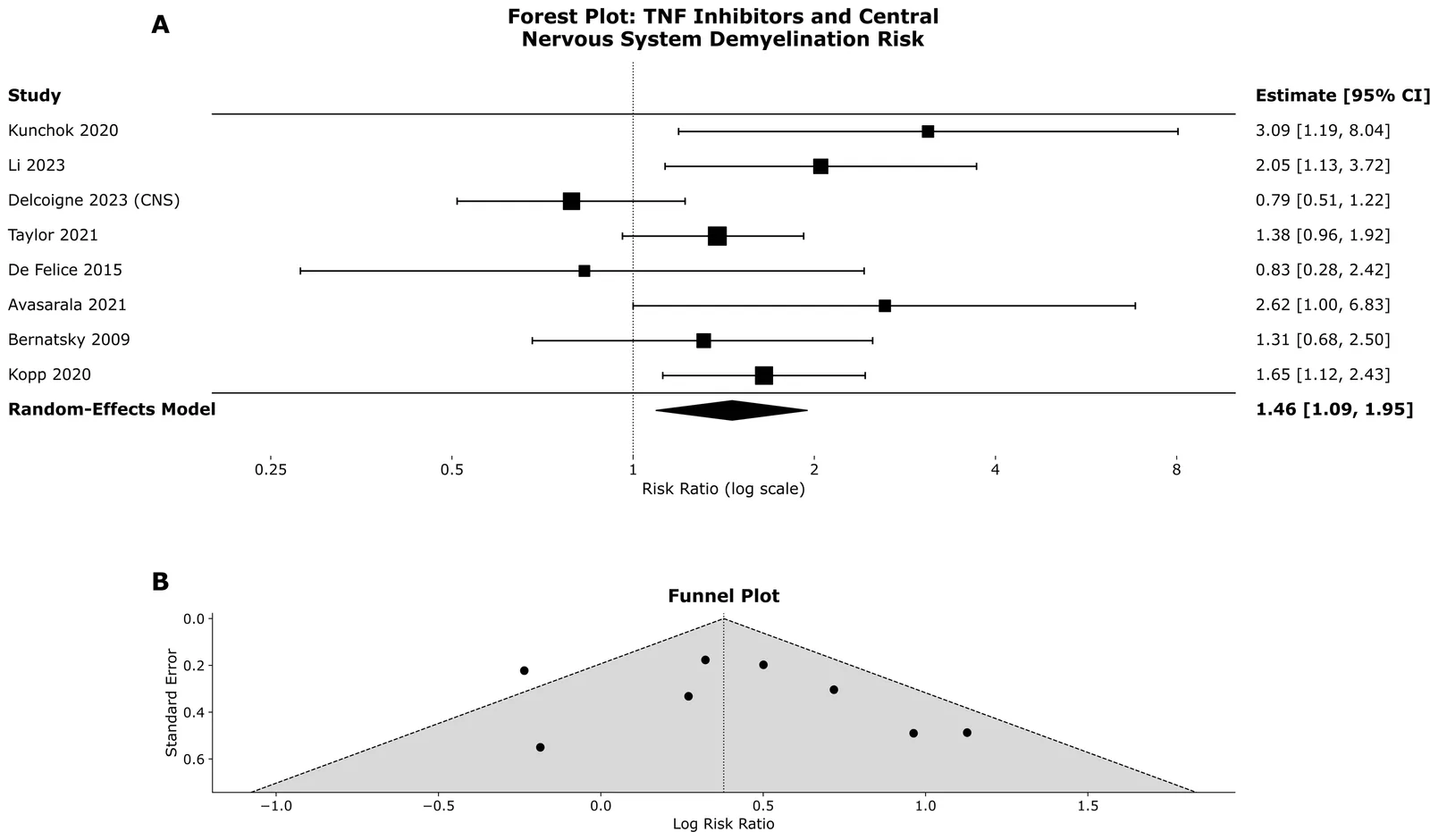
Forest plot and funnel plot for tumor necrosis factor alpha inhibitor (TNFi) exposure and central nervous system (CNS) demyelination risk. (A) Forest plot showing individual study risk ratios and the pooled effect estimate for the association between TNF inhibitor exposure and central nervous system demyelinating disorders [19-22,25,26,28,30]. (B) Funnel plot assessing publication bias for the CNS demyelination meta-analysis.

PNS Demyelination Subgroup Analysis

Three studies evaluating PNS demyelinating outcomes were included in the PNS subgroup meta-analysis. Moderate heterogeneity was observed (tau² = 0.1015; I² = 47.4%; H² = 1.90), indicating moderate variability among studies. The pooled analysis demonstrated an increased risk of peripheral demyelinating disorders associated with TNF inhibitor exposure (pooled RR 2.00, 95% CI 1.19-3.38), although heterogeneity between studies was lower than that observed in the CNS subgroup. These findings are illustrated in the subgroup forest plot (Figure 4A).

**Figure 4:**
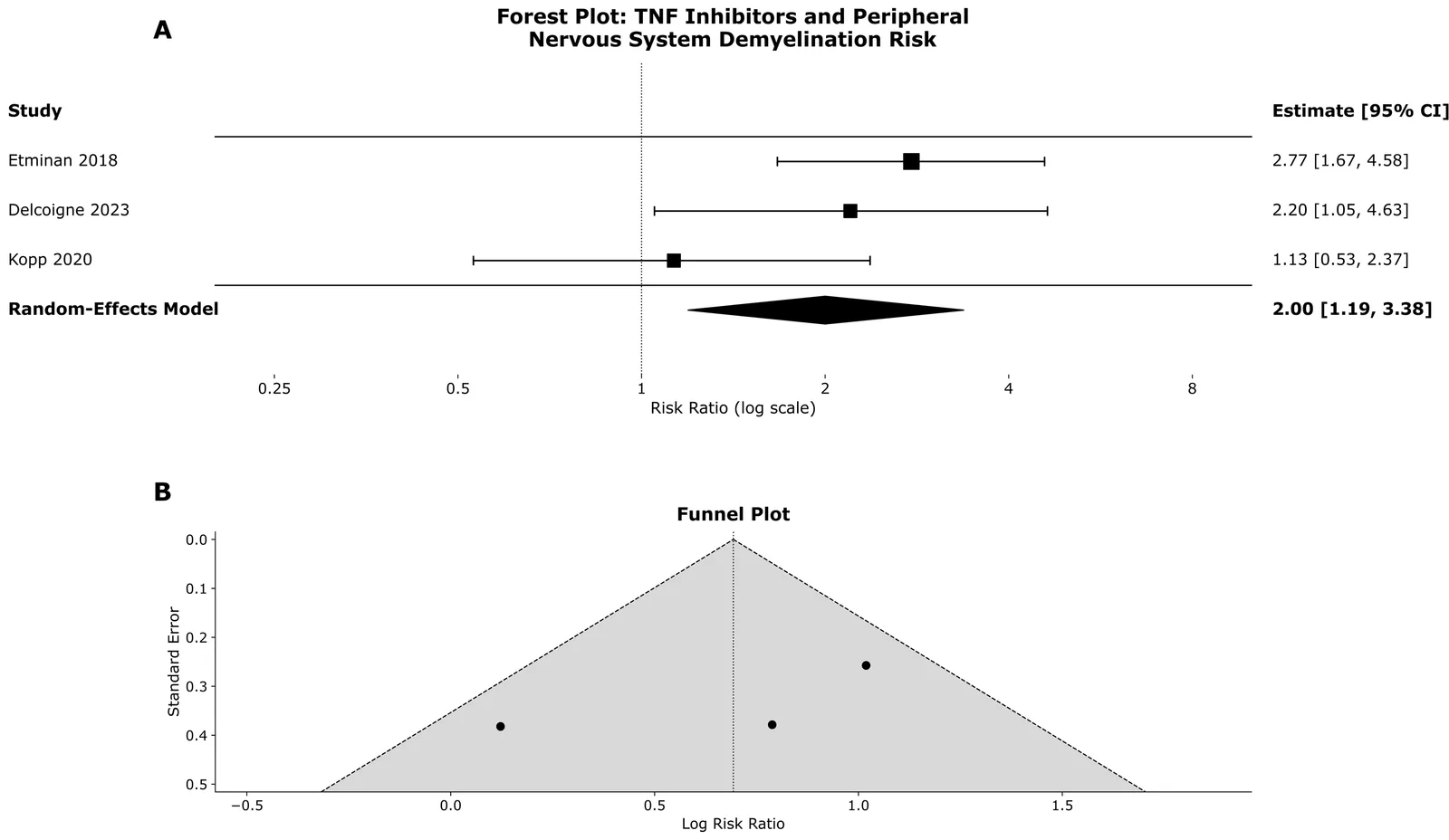
Forest plot and funnel plot for tumor necrosis factor alpha inhibitor (TNFi) exposure and peripheral nervous system (PNS) demyelination risk. (A) Forest plot showing individual study risk ratios and the pooled effect estimate for the association between TNF inhibitor exposure and peripheral nervous system demyelinating disorders [25-27]. (B) Funnel plot assessing publication bias for the PNS demyelination meta-analysis.

Subgroup Comparison Between CNS and PNS Outcomes

Subgroup analysis using mixed-effects meta-regression demonstrated no statistically significant difference between CNS and PNS demyelination risk associated with TNF inhibitor exposure (QM = 0.94, p = 0.334). This suggests that TNF inhibitor exposure may be associated with increased risk of demyelination affecting both CNS and PNS without a statistically significant difference in magnitude between these outcome types (Table 2). Given the small number of studies contributing to each subgroup (k = 8 for CNS and k = 3 for PNS), this comparison is underpowered, and the absence of a statistically significant difference should be interpreted as inconclusive rather than as evidence that the two risks are equivalent.

**Table 2:** Meta-analysis heterogeneity, model parameters, and publication bias assessment.

Analysis	k (studies)	tau²	tau	I² (%)	H²	Q (df)	Q p-value	Egger z	Egger p-value
Overall	11	0.0995	0.3154	54.2	2.18	21.85 (10)	0.0159	0.76	0.449
Central nervous system demyelination	8	0.0807	0.2840	50.4	2.01	14.10 (7)	0.0494	0.78	0.437
Peripheral nervous system demyelination	3	0.1015	0.3186	47.4	1.90	3.80 (2)	0.1495	-1.27	0.205
Subgroup difference	-	-	-	-	-	QM = 0.94	0.334	-	-

Publication Bias Assessment

Publication bias was evaluated using funnel plot analysis and Egger’s regression test. Funnel plot inspection did not reveal substantial asymmetry (Figure 3B and Figure 4B). Egger’s regression test did not demonstrate statistically significant evidence of publication bias in the overall meta-analysis (z = 0.76, p = 0.449), the CNS subgroup (z = 0.78, p = 0.437), or the PNS subgroup (z = -1.27, p = 0.205). However, Egger’s regression test has limited reliability when fewer than 10 studies are available; therefore, these findings should be interpreted cautiously. In particular, the PNS subgroup result (k = 3) should be considered exploratory rather than a reliable indicator of the presence or absence of publication bias.

Sensitivity Analysis

Sensitivity analyses using leave-one-out procedures were performed for both the overall (k = 11) and CNS (k = 8) analyses; full per-study results are reported in Appendix 2. For the overall analysis, excluding any single study left the pooled estimate within the range RR 1.47-1.74, and the association remained statistically significant in all eleven leave-one-out iterations (95% CI lower bounds 1.14-1.40). For the CNS subgroup, excluding any single study left the pooled estimate within the range RR 1.37-1.59, and the association likewise remained statistically significant in all eight leave-one-out iterations (95% CI lower bounds 1.01-1.29); excluding Delcoigne et al. [25] (CNS) reduced heterogeneity from I² = 50.4% to I² = 0.1%, identifying this study as the principal driver of between-study heterogeneity in the CNS subgroup, though it did not change the direction or significance of the pooled effect. The 95% prediction interval for a new study's true effect was 0.66-3.19 for the CNS subgroup and 0.73-3.46 overall; both intervals cross the null, indicating that while the average effect is robust to the removal of any single study, the effect in a new, as-yet-unstudied population cannot be predicted with the same confidence. A prediction interval was not calculated for the PNS subgroup because k = 3 (df = 1) is too few studies for a meaningful estimate. Because the 11 included studies reported five different effect-measure types (see Statistical Analysis section and Appendix 2), a sensitivity analysis restricted to the three studies reporting an RR directly [22,27,30] was performed. This RR-only subset yielded a pooled RR of 1.61 (95% CI 0.81-3.19; I² = 64.7%), directionally consistent with the full 11-study pooled estimate (RR 1.59, 95% CI 1.22-2.08), though with a wider confidence interval reflecting the smaller sample. Pooled estimates stratified by measure type are reported in Appendix 2; the HR-based subset (k = 4, pooled RR 1.30, 95% CI 0.82-2.07) includes the two estimates derived from an active TNFi-vs-TNFi comparator (oTNFi vs etanercept [25]) rather than a TNFi-vs-unexposed comparator, and should be interpreted as answering a related but distinct clinical question.

Discussion

This systematic review and meta-analysis evaluated the association between TNFi exposure and the risk of demyelinating disorders affecting both the CNS and PNS. A total of 13 observational studies were included in the systematic review, with 11 studies providing sufficient quantitative data for meta-analysis. The pooled analysis demonstrated an increased risk of demyelinating disorders among patients receiving TNF inhibitors, with elevated risk estimates observed for both CNS and PNS outcomes [19-22,25,28]. Subgroup analysis showed that TNF inhibitor exposure was associated with increased risk of demyelination in both neurological compartments, although the difference in magnitude between CNS and PNS subgroups was not statistically significant (p = 0.334), likely reflecting the limited number of studies available for each subgroup rather than true equivalence of risk. These findings suggest that TNF inhibition may contribute to demyelinating processes through mechanisms that affect both central and PNS structures [8,10,32].

The results of this meta-analysis are consistent with prior observational studies, pharmacovigilance reports, and registry-based cohort analyses that have suggested an association between TNF inhibitor therapy and demyelinating disorders [33-35]. Several large population-based studies conducted in North America and Europe have reported increased incidence of MS, ON, and inflammatory neuropathies in patients exposed to TNF inhibitors compared to unexposed populations [19-21,25,28]. These findings have raised concerns regarding the neurological safety profile of TNF inhibitors, particularly in patients with autoimmune diseases who may already have altered immune regulation [5,6,8]. The present meta-analysis strengthens the existing evidence by providing pooled quantitative estimates derived from large observational cohorts and registry datasets, and by demonstrating that the risk extends beyond CNS demyelination to include PNS demyelinating disorders [23,24].

The biological plausibility of TNFi-associated demyelination is supported by the complex role of tumor necrosis factor in immune regulation and neural homeostasis [36,37]. TNF exerts both pro-inflammatory and neuroprotective effects through its interaction with two distinct receptors, TNFR1 and TNFR2 [37,38]. While TNFR1 signaling promotes inflammatory responses and apoptosis, TNFR2 plays a critical role in neuroprotection, oligodendrocyte survival, and remyelination [37]. Inhibition of TNF may disrupt TNFR2-mediated protective pathways, impair remyelination processes, and increase susceptibility to immune-mediated demyelination [10]. Experimental studies have demonstrated that TNF inhibition can worsen demyelinating disease in animal models, and clinical observations have reported new-onset demyelination or exacerbation of pre-existing demyelinating disorders following TNF inhibitor therapy [7,10,35,39]. Furthermore, TNFi may alter immune surveillance and regulatory T-cell function, potentially promoting autoimmune responses directed against myelin structures [40,41]. The involvement of both CNS and PNS demyelination observed in this analysis supports the hypothesis that TNF inhibition affects systemic immune mechanisms rather than being limited to a specific neurological compartment [9,42].

Moderate heterogeneity was observed across studies in both CNS (I² = 50.4%) and PNS (I² = 47.4%) subgroup analyses. This heterogeneity likely reflects differences in study populations, underlying autoimmune conditions, TNF inhibitor types, treatment duration, follow-up periods, and outcome definitions [19-21,25]. Registry-based studies included diverse patient populations across different healthcare systems, which may contribute to variability in risk estimates [20,21,28]. Differences in baseline neurological risk factors, disease severity, and concurrent immunosuppressive therapies may also influence demyelination risk [43,44]. Despite this heterogeneity, the overall direction of effect was consistent across studies, supporting the robustness of the observed association. Sensitivity analyses demonstrated that no individual study disproportionately influenced the pooled effect estimate, further supporting the stability and reliability of the findings.

Risk-of-bias assessment using the ROBINS-I tool demonstrated that most included studies were of acceptable methodological quality, with low risk of bias in domains related to intervention classification, outcome measurement, and missing data. Moderate risk of bias was observed primarily in the confounding domain, which is expected in observational studies where treatment allocation is not randomized [19-21]. However, many included studies were large population-based cohorts with robust exposure and outcome ascertainment using national registries, which enhances the reliability of the findings [25,28]. Risk-of-bias visualization using robvis confirmed that most studies with an active comparator group had low-to-moderate overall risk, largely attributable to confounding inherent to observational designs (Appendix 3).

Publication bias assessment using funnel plots and Egger’s regression test did not demonstrate statistically significant asymmetry, suggesting a low likelihood of small-study effects or selective publication [19,25]. Egger’s test was not statistically significant in either the overall analysis or the PNS subgroup. However, the relatively small number of studies included in the PNS subgroup limits the statistical power of publication bias detection, and these findings should be interpreted cautiously [23,24]. Nonetheless, the inclusion of large registry-based cohort studies reduces the likelihood that the observed association is driven solely by publication bias.

The clinical implications of these findings are important for the management of patients receiving TNF inhibitor therapy. TNF inhibitors are highly effective treatments for a range of autoimmune diseases, including RA, IBD, AS, and PsA [2,3,5]. While the absolute risk of demyelinating disorders remains low, clinicians should be aware of the potential neurological risks associated with TNF inhibition [9,32,45]. Careful neurological assessment prior to initiation of TNFi therapy and ongoing monitoring during treatment may help identify early neurological symptoms [43,44]. Patients with pre-existing demyelinating disorders or strong family history of demyelination may require alternative treatment strategies [20,44]. Early recognition of neurological symptoms may allow prompt discontinuation of TNFi and appropriate neurological management [10,45].

This study has several strengths. It included a comprehensive literature search covering multiple major databases over a 26-year period and incorporated large population-based cohort and registry studies with substantial sample sizes [19-21,25,28]. Risk of bias was systematically assessed using the ROBINS-I tool, and visualization was performed using standardized robvis methodology [17,18]. The use of random-effects meta-analysis allowed appropriate modeling of between-study heterogeneity. Subgroup analyses provided important insights into differences between CNS and PNS demyelination risk. Statistical analyses were performed using established meta-analytic methods in RStudio, ensuring reproducibility and methodological rigor.

Several limitations should also be considered. First, all included studies were observational, and residual confounding cannot be completely excluded. Patients receiving TNFi may differ from unexposed patients in ways that influence demyelination risk [19-21]. Second, moderate heterogeneity was present across studies, reflecting differences in study design and populations [25,28]. Third, variation in TNFi types and treatment duration may influence risk estimates but could not be fully evaluated due to limited subgroup data [7,23]. Fourth, the limited number of PNS studies reduces statistical power for subgroup analysis and publication bias assessment [23,24]. Fifth, some Nordic registry-based studies (e.g., Delcoigne et al. [25] and Kopp et al. [26]) draw on overlapping national registers and may share underlying patients, which could lead to underestimation of the pooled variance if the same individuals contributed to more than one pooled estimate; this could not be fully verified from the published reports. Sixth, differences in diagnostic criteria and registry reporting may introduce variability in outcome ascertainment [33,34].

The implications for clinical practice are important. These findings support cautious use of TNFi in patients with a personal or family history of demyelinating disorders and emphasize the importance of neurological monitoring during treatment, particularly in higher-risk individuals. Alternative therapies should be considered when TNFi-related demyelination is suspected. Further research is needed to identify relevant risk factors, genetic predispositions, and long-term neurological outcomes associated with TNFi use.

## Conclusions

In conclusion, this systematic review and meta-analysis demonstrates that TNFi exposure is associated with an increased risk of demyelinating disorders affecting both the CNS and PNS. These findings highlight the importance of careful neurological monitoring in patients receiving TNFi. Although the absolute risk remains low, clinicians should remain vigilant for neurological symptoms, particularly in patients with underlying neurological susceptibility. Future prospective studies, mechanistic investigations, and drug-specific safety analyses are needed to further clarify the relationship between TNF inhibition and demyelination risk and to identify patient populations at highest risk. Personalized treatment approaches and ongoing surveillance will be important to mitigate these potential neurological risks.

## Appendices

 Appendix 1

**Table 3:** Complete database search strategies.

#	Search string
1	"Tumor Necrosis Factor-alpha"[Mesh] OR "Tumor Necrosis Factor Inhibitors"[Mesh]
2	(tumor necrosis factor inhibitor*[tiab] OR TNF inhibitor*[tiab] OR TNF-alpha inhibitor*[tiab] OR anti-TNF*[tiab] OR TNF antagonist*[tiab] OR TNF blocker*[tiab] OR biologic therapy[tiab] OR biologic agent*[tiab] OR biological DMARD*[tiab])
3	(infliximab[tiab] OR adalimumab[tiab] OR etanercept[tiab] OR golimumab[tiab] OR certolizumab[tiab] OR certolizumab pegol[tiab])
4	#1 OR #2 OR #3
5	"Demyelinating Diseases"[Mesh] OR "Multiple Sclerosis"[Mesh] OR "Optic Neuritis"[Mesh] OR "Guillain-Barre Syndrome"[Mesh] OR "Polyradiculoneuropathy, Chronic Inflammatory Demyelinating"[Mesh]
6	(demyelinat*[tiab] OR "multiple sclerosis"[tiab] OR "optic neuritis"[tiab] OR "Guillain-Barre syndrome"[tiab] OR "Guillain-Barre"[tiab] OR "inflammatory neuropathy"[tiab] OR "inflammatory polyneuropathy"[tiab] OR "chronic inflammatory demyelinating polyneuropathy"[tiab] OR CIDP[tiab] OR "transverse myelitis"[tiab] OR "peripheral neuropathy"[tiab])
7	#5 OR #6
8	#4 AND #7

#	Search string
1	'tumor necrosis factor alpha inhibitor'/exp
2	(tumor NEXT/1 necrosis NEXT/1 factor NEXT/1 inhibitor* OR 'tnf inhibitor*' OR 'tnf-alpha inhibitor*' OR 'anti-tnf*' OR 'tnf antagonist*' OR 'tnf blocker*' OR 'biologic therapy' OR 'biological dmard*'):ti,ab,kw
3	'infliximab'/exp OR 'adalimumab'/exp OR 'etanercept'/exp OR 'golimumab'/exp OR 'certolizumab pegol'/exp OR (infliximab OR adalimumab OR etanercept OR golimumab OR certolizumab):ti,ab,kw
4	#1 OR #2 OR #3
5	'demyelinating disease'/exp OR 'multiple sclerosis'/exp OR 'optic neuritis'/exp OR 'Guillain Barre syndrome'/exp OR 'chronic inflammatory demyelinating polyneuropathy'/exp
6	(demyelinat* OR 'multiple sclerosis' OR 'optic neuritis' OR 'guillain-barre syndrome' OR 'guillain barre' OR 'inflammatory neuropathy' OR 'inflammatory polyneuropathy' OR cidp OR 'transverse myelitis' OR 'peripheral neuropathy'):ti,ab,kw
7	#5 OR #6
8	#4 AND #7

#	Search string
1	TITLE-ABS-KEY("tumor necrosis factor inhibitor*" OR "TNF inhibitor*" OR "TNF-alpha inhibitor*" OR "anti-TNF*" OR "TNF antagonist*" OR "TNF blocker*" OR "biologic therapy" OR "biological DMARD*" OR infliximab OR adalimumab OR etanercept OR golimumab OR certolizumab)
2	TITLE-ABS-KEY(demyelinat* OR "multiple sclerosis" OR "optic neuritis" OR "Guillain-Barre syndrome" OR "Guillain Barre" OR "inflammatory neuropathy" OR "inflammatory polyneuropathy" OR "chronic inflammatory demyelinating polyneuropathy" OR CIDP OR "transverse myelitis" OR "peripheral neuropathy")
3	#1 AND #2

#	Search string
1	MeSH descriptor: [Tumor Necrosis Factor-alpha] explode all trees
2	(tumor necrosis factor inhibitor* OR TNF inhibitor* OR TNF-alpha inhibitor* OR anti-TNF* OR TNF antagonist* OR TNF blocker* OR biologic therapy OR biological DMARD*):ti,ab,kw
3	(infliximab OR adalimumab OR etanercept OR golimumab OR certolizumab):ti,ab,kw
4	#1 OR #2 OR #3
5	MeSH descriptor: [Demyelinating Diseases] explode all trees OR MeSH descriptor: [Multiple Sclerosis] explode all trees OR MeSH descriptor: [Optic Neuritis] explode all trees OR MeSH descriptor: [Guillain-Barre Syndrome] explode all trees
6	(demyelinat* OR "multiple sclerosis" OR "optic neuritis" OR "Guillain-Barre syndrome" OR "inflammatory neuropathy" OR "inflammatory polyneuropathy" OR "chronic inflammatory demyelinating polyneuropathy" OR CIDP OR "transverse myelitis" OR "peripheral neuropathy"):ti,ab,kw
7	#5 OR #6
8	#4 AND #7

Appendix 2 

**Table 4:** Per-study effect estimates, pooled analyses, and leave-one-out sensitivity analyses.

Study	Subgroup	Effect measure	Estimate	95% CI lower	95% CI upper	log (estimate)	SE [log(estimate)]	Variance [log(estimate)]	Weight in subgroup pooled model (%)	Weight in overall pooled model (%)
Kunchok et al. [19], 2020	CNS	OR	3.09	1.19	8.04	1.1282	0.4874	0.2375	7	5.5
Li et al. [21], 2023	CNS	IRR	2.05	1.13	3.72	0.7178	0.304	0.0924	12.9	9.6
Delcoigne et al. [25], 2023 (CNS)	CNS	HR	0.79	0.51	1.22	-0.2357	0.2225	0.0495	17.1	12.4
Taylor et al. [20], 2021	CNS	SIR	1.38	0.96	1.92	0.3221	0.1768	0.0313	19.9	14.1
De Felice et al. [22], 2015	CNS	RR	0.83	0.28	2.42	-0.1863	0.5502	0.3027	5.8	4.6
Avasarala et al. [28], 2021	CNS	IRR	2.62	1	6.83	0.9632	0.4901	0.2402	6.9	5.4
Bernatsky et al. [30], 2009	CNS	RR	1.31	0.68	2.5	0.27	0.3321	0.1103	11.7	8.8
Kopp et al. [26], 2020 (CNS)	CNS	HR	1.65	1.12	2.43	0.5008	0.1976	0.039	18.6	13.3
Etminan et al. [27], 2018	PNS	RR	2.77	1.67	4.58	1.0188	0.2574	0.0662	42.3	11.1
Delcoigne et al. [25], 2023 (PNS)	PNS	HR	2.2	1.05	4.63	0.7885	0.3785	0.1433	29	7.6
Kopp et al. [26], 2020 (PNS)	PNS	HR	1.13	0.53	2.37	0.1222	0.3821	0.146	28.7	7.5

Analysis	k	Pooled RR	95% CI lower	95% CI upper	tau²	tau	I² (%)	H²	Q	df	Q p-value	Egger z	Egger p-value	95% prediction interval
Overall (all 11 studies)	11	1.59	1.22	2.08	0.0995	0.3154	54.2	2.18	21.85	10	0.0159	0.76	0.449	0.73-3.46
CNS demyelination subgroup	8	1.46	1.09	1.95	0.0807	0.284	50.4	2.01	14.1	7	0.0494	0.78	0.437	0.66-3.19
PNS demyelination subgroup	3	2	1.19	3.38	0.1015	0.3186	47.4	1.9	3.8	2	0.1495	-1.27	0.205	Not meaningful (k = 3, df = 1)
Sensitivity: RR-measure studies only	3	1.61	0.81	3.19	-	-	64.7	-	-	-	-	-	-	-
Sensitivity: HR-measure studies only	4	1.3	0.82	2.07	-	-	64.7	-	-	-	-	-	-	-
Sensitivity: IRR-measure studies only	2	2.19	1.32	3.64	-	-	0	-	-	-	-	-	-	-

Analysis	Study excluded	Pooled RR	95% CI lower	95% CI upper	I² (%)
CNS subgroup (k = 8)	Kunchok et al. [19], 2020	1.37	1.03	1.83	47.2
CNS subgroup (k = 8)	Li et al. [21], 2023	1.39	1.01	1.9	51.4
CNS subgroup (k = 8)	Delcoigne et al. [25], 2023 (CNS)	1.59	1.29	1.96	0.1
CNS subgroup (k = 8)	Taylor et al. [20], 2021	1.5	1.03	2.18	57.4
CNS subgroup (k = 8)	De Felice et al. [22], 2015	1.51	1.11	2.05	54.5
CNS subgroup (k = 8)	Avasarala et al. [28], 2021	1.39	1.03	1.88	51.6
CNS subgroup (k = 8)	Bernatsky et al. [30], 2009	1.49	1.07	2.08	57.3
CNS subgroup (k = 8)	Kopp et al. [26], 2020 (CNS)	1.43	1.01	2.04	54.5
Overall (k = 11)	Kunchok et al. [19], 2020	1.53	1.17	2	54.3
Overall (k = 11)	Li et al. [21], 2023	1.55	1.16	2.07	56.8
Overall (k = 11)	Delcoigne et al. [25], 2023 (CNS)	1.74	1.4	2.16	22.4
Overall (k = 11)	Taylor et al. [20], 2021	1.63	1.2	2.24	58
Overall (k = 11)	De Felice et al. [22], 2015	1.64	1.25	2.16	56.3
Overall (k = 11)	Avasarala et al. [28], 2021	1.55	1.17	2.04	56.3
Overall (k = 11)	Bernatsky et al. [30], 2009	1.63	1.21	2.17	58.4
Overall (k = 11)	Kopp et al. [26], 2020 (CNS)	1.59	1.17	2.17	58.4
Overall (k = 11)	Etminan et al. [27], 2018	1.47	1.14	1.9	43.1
Overall (k = 11)	Delcoigne et al. [25], 2023 (PNS)	1.55	1.17	2.06	56.9
Overall (k = 11)	Kopp et al. [26], 2020 (PNS)	1.64	1.23	2.18	57.5

Appendix 3 

**Table 5:** Risk Of Bias In Non-randomized Studies - of Interventions (ROBINS-I) risk-of-bias assessment.

Study	Design	All judgements (compiled)	D1 Judgement	D1 Rationale	D2 Judgement	D2 Rationale	D3 Judgement	D3 Rationale	D4 Judgement	D4 Rationale	D5 Judgement	D5 Rationale	D6 Judgement	D6 Rationale	D7 Judgement	D7 Rationale	Overall judgement
Kunchok et al. [19], 2020	Nested case-control (Mayo Clinic, USA)	D1: Moderate | D2: Low | D3: Low | D4: Low | D5: Low | D6: Low | D7: Low	Moderate	Matched by birth year, sex, and disease type, with adjustment for disease duration; residual confounding by indication is possible but partly mitigated by drawing controls from the same DMT-treated population.	Low	Controls drawn from the same source population and matched 1:1 with defined follow-up/survival requirements; the high exclusion rate on chart review reflects application of clear, pre-defined eligibility criteria rather than outcome-related selection.	Low	TNFi exposure ascertained via full medical-record review with clear, time-anchored definitions, independent of outcome status.	Low	No specific evidence of differential co-therapy or non-adherence between groups is reported; standard for this observational design.	Low	Missing exposure-timing data were small (2-9%) and the primary case-control analysis was essentially complete.	Low	Outcomes confirmed via structured chart review against explicit, objective diagnostic criteria (2017 McDonald, 2015 NMOSD), applied identically to both groups.	Low	Primary and named secondary analyses are all reported, including a null finding, consistent with transparent reporting.	Moderate
Li et al. [21], 2023	Nested case-control (Canada, population registry)	D1: Moderate | D2: Low | D3: Low | D4: Low | D5: Low | D6: Low | D7: Low	Moderate	Adjustment via matching plus sex and Charlson Comorbidity Index; smoking/lifestyle factors unmeasured but partially addressed via a reported E-value sensitivity check.	Low	Population-based, density-sampled design with prior-diagnosis exclusion and a registration run-in period, minimizing prevalent-user/immortal-time bias.	Low	Exposure defined from pharmacy dispensation records - an objective, non-recall-based measure.	Low	Dispensation-based exposure ascertainment is the field-standard approach for claims/registry pharmacoepidemiology and is not a study-specific flaw.	Low	Closed administrative cohort with negligible loss to follow-up and comparable follow-up duration between groups.	Low	MS ascertained via a previously validated algorithm (PPV 99.5%) applied identically regardless of exposure status.	Low	Multiple prespecified sensitivity analyses (1-year window, latency period) reported consistently, including non-significant results.	Moderate
Delcoigne et al. [25], 2023	Cohort (Nordic clinical registers, new-user design)	D1: Moderate | D2: Low | D3: Low | D4: Low | D5: Low | D6: Low | D7: Low	Moderate	Adjusted for age, sex, calendar period, disease duration, CRP, and concomitant methotrexate; smoking could not be adjusted for, a limitation the authors acknowledge directly.	Low	New-user (incident treatment initiator) design with exclusion of patients with prior neuroinflammatory events, avoiding prevalent-user bias.	Low	Exposure from prospectively registered TNFi treatment initiation with clear handling rules, recorded independent of outcome.	Low	Primary analysis uses an ITT-like approach with an on-drug sensitivity analysis showing only minor differences, indicating robustness to this concern.	Low	Register-linkage follow-up with minimal loss to follow-up; missing CRP was handled via a missing-indicator category rather than left unaddressed.	Low	Outcomes from linked National Patient Register ICD-10 diagnoses, a standard, previously used ascertainment approach for this data source, applied equally in both groups.	Low	Four outcome definitions and several predefined sensitivity analyses were all reported regardless of direction or significance.	Moderate
Taylor et al. [20], 2021	Prospective cohort (UK BSRBR-RA registry)	D1: Moderate | D2: Low | D3: Low | D4: Low | D5: Low | D6: Low | D7: Low	Moderate	Rich baseline covariates recorded, but the design compares against external population rates rather than an adjusted active comparator; a genuine but field-typical limitation for a pharmacovigilance registry.	Low	Registration required at TNFi start with no known prior demyelination; the authors' candid note about possible clinician risk-avoidance reflects appropriate transparency rather than a design flaw that was left unaddressed.	Low	Anti-TNFα initiation/drug/dates prospectively recorded by treating rheumatologists, with outcome classification performed by a neurologist blinded to treatment details.	Low	Switches and cessation were tracked, with an on/off-therapy analysis included.	Low	Very large registry follow-up (172,107 person-years); some ancillary data gaps are explicitly and transparently flagged by the authors rather than silently handled.	Low	Centralized, blinded neurologist review using McDonald-informed criteria is a relatively rigorous ascertainment method for a pharmacovigilance registry.	Low	Prespecified outcomes all reported regardless of direction, including a non-significant primary result.	Moderate
De Felice et al. [22], 2015	Retrospective cohort (single tertiary center, USA)	D1: Moderate | D2: Moderate | D3: Low | D4: Low | D5: Moderate | D6: Low | D7: Low	Moderate	No multivariable adjustment was performed, but relevant covariates were measured and tabulated, and every case underwent uniform, criteria-based diagnostic confirmation, which offsets some of the concern.	Moderate	Single-center cohort with post hoc case winnowing on review; mitigated somewhat by explicit, consistently applied confirmation criteria for both exposed and unexposed suspected cases.	Low	Exposure defined via structured search terms for four named agents, ascertained independently of the separately confirmed outcome.	Low	No specific evidence of differential co-therapy patterns undermining comparability is reported beyond what is expected in this design.	Moderate	Follow-up duration for suspected cases differed notably between exposed and unexposed groups, a specific, quantified imbalance the paper does not further address.	Low	Outcome confirmed against objective 2010 McDonald MRI criteria by a single MS-subspecialty neurologist, applied consistently to both groups.	Low	IBD/CD/UC-stratified analyses were pre-specified and reported consistently throughout.	Moderate
Avasarala et al. [34], 2021	Retrospective cohort (US claims database)	D1: Moderate | D2: Low | D3: Low | D4: Low | D5: Low | D6: Moderate | D7: Low	Moderate	Adjustment limited to age and sex, a real gap, but the very large sample (>1 million patients) and matched-control design provide some offsetting stability, and the authors are transparent about this limitation.	Low	Clearly defined enrollment windows and 4:1 matched controls are a reasonable design for a claims-based cohort of this scale.	Low	Exposure defined from pharmacy claims for four named agents, classified independently of and prior to outcome.	Low	Standard claims-database limitation (no adherence data); not a study-specific design flaw.	Low	Very large, clearly defined cohort with complete event/person-time accounting in the reported tables.	Moderate	Claims-based MS algorithm applied identically to both groups, though the authors themselves note claims data cannot be validated against records, and monophasic events were excluded from the outcome definition.	Low	Overall and subgroup analyses (UC/CD) reported with both unadjusted and adjusted estimates regardless of significance.	Moderate
Bernatsky et al. [36], 2009	Nested case-control (Canada, administrative database)	D1: Moderate | D2: Moderate | D3: Moderate | D4: Moderate | D5: Moderate | D6: Moderate | D7: Moderate	Moderate	Adjusted for age, a severity proxy (visit frequency), and a full panel of concomitant medications; disease duration/severity was not directly measured but partially addressed via the visit-frequency proxy.	Moderate	The authors identify and directly correct for a channeling-bias pattern via stratified sensitivity analysis, which is a transparent and appropriate methodological response to a genuine but disclosed limitation.	Moderate	Exposure defined objectively from pharmacy dispensing data, independent of outcome knowledge, though based on dispensing rather than confirmed intake.	Moderate	Concomitant medications were measured and adjusted for, offsetting some concern about the documented switching pattern.	Moderate	Full case-control accounting reported; short average follow-up (1.9 years) is typical for this administrative-database design.	Moderate	Outcome ascertainment via administrative codes is a real limitation the authors themselves probe and partially validate via cross-database comparison, showing awareness and partial mitigation rather than an unexamined flaw.	Moderate	Multiple drug classes and estimates are transparently reported, including how results changed under stratification.	Moderate
Kopp et al. [26], 2019	Cohort (Danish DANBIO / Swedish ARTIS registries)	D1: Moderate | D2: Low | D3: Low | D4: Low | D5: Low | D6: Low | D7: Low	Moderate	Adjusted for gender and calendar year in the primary model, with disease-activity adjustment in registry-specific sensitivity analyses that the authors report did not materially change estimates.	Low	Prospective, population-based registers with near-complete coverage and exclusion of patients with pre-existing neuroinflammatory diagnoses.	Low	Exposure defined objectively and prospectively (time-updated) from clinical/biologics registers, independent of outcome.	Low	Time-dependent exposure modeling with an on-drug sensitivity analysis supports robustness to co-intervention/discontinuation concerns.	Low	Very large, long-follow-up cohort (1.38 million person-years) via linked national registers with minimal loss to follow-up for the core exposure/outcome variables.	Low	Outcomes via validated ICD-10 codes (96.3% MS validity reported) applied identically to both groups.	Low	Broad pre-specified outcome and sensitivity analyses all reported transparently regardless of direction.	Moderate
Etminan et al. [27], 2018	Nested case-control (US claims database)	D1: Moderate | D2: Low | D3: Low | D4: Low | D5: Low | D6: Moderate | D7: Low	Moderate	Adjusted for eight relevant covariates plus a methotrexate+DMARD comparator specifically chosen to address confounding by indication; residual confounding is acknowledged but meaningfully addressed by design.	Low	Density-based incidence sampling with a new-user design explicitly used to avoid prevalent-user bias, and prior neuropathy excluded at entry.	Low	Exposure from pharmacy dispensing with clear, outcome-independent risk windows.	Low	A dose-response analysis (2 vs 3+ prescriptions) showing a gradient supports the exposure-outcome relationship despite the standard dispensation-only limitation.	Low	Complete captured data by construction, with comparable mean follow-up between cases and controls.	Moderate	PN ascertained via ICD-9/10 codes selected by a neurologist co-author; the authors' transparency about lacking chart access is a genuine limitation but the code selection itself was clinically informed.	Low	Multiple exposure comparators and windows are reported with full estimates across tables.	Moderate
Tsouni et al. [23], 2015	Single-arm retrospective case series (Switzerland)	D1: Serious | D2: Moderate | D3: Low | D4: Moderate | D5: Moderate | D6: Low | D7: Low	Serious	No unexposed comparator group exists, so an unconfounded risk estimate cannot be derived from this design alone; softened from Critical because the study still provides clinically useful descriptive evidence on TNFi-associated neuropathy phenotype and course.	Moderate	Cases identified via a systematic ICD-10 plus free-text keyword chart search among all patients who underwent NCS/EMG in the relevant period, a reasonably systematic (if retrospective) case-finding approach.	Low	Exposure to four named agents was well-documented via pharmacy/chart records with start dates and duration.	Moderate	No comparator arm exists to assess co-intervention differences, though the case series itself reports exposure and concomitant treatment consistently.	Moderate	Some patients were lost to follow-up for the underlying-disease outcome, a limitation the authors themselves disclose transparently.	Low	Rigorous multi-modal outcome ascertainment (EFNS/PNS 2010 criteria, MRI, CSF, biopsy in select patients).	Low	A single pre-specified outcome (neuropathy phenotype/course) is reported without evidence of selective alternative-outcome reporting.	Serious
Ekinci et al. [24], 2021	Case-control (single center, Turkey)	D1: Moderate | D2: Low | D3: Low | D4: Low | D5: Low | D6: Low | D7: Moderate	Moderate	Comparator is a healthy population rather than a TNFi-naive disease-matched group, a real limitation partly offset by explicit exclusion of confounding comorbidities (diabetes, endocrine/neurological disease) from both groups.	Low	Defined 1-year recruitment window with clearly stated, symmetric exclusion criteria aimed at removing neuropathy mimics.	Low	Exposure well-characterized by agent and duration across the 35 TNFi-treated patients.	Low	No specific evidence of problematic co-therapy differences is reported.	Low	All enrolled patients and controls completed examination and EMG with no reported dropouts.	Low	Nerve conduction measured via a standardized EMG protocol by a specialist neurologist in both groups.	Moderate	Numerous nerve-conduction parameters were compared without correction for multiple comparisons; a genuine, specific limitation in how the significant subset is emphasized.	Moderate
Lucchini et al. [7], 2021	Single-arm retrospective case series (Italy)	D1: Serious | D2: Serious | D3: Moderate | D4: Low | D5: Moderate | D6: Low | D7: Low	Serious	No comparator group exists; softened from Critical because the study contributes valid, well-characterized descriptive case evidence to the narrative synthesis even though it cannot support a standalone causal estimate.	Serious	Cases were identified by searching a specialized demyelinating-disorders clinic database, meaning only patients who already had a qualifying CNS event could enter - an outcome-dependent sampling frame inherent to this design and not fully avoidable via more careful conduct.	Moderate	Exposure (agent, duration, temporal window) was ascertained from chart review with reasonable detail, applied consistently across all 10 cases.	Low	No evidence of problematic deviations is reported; all patients received defined TNFi monotherapy regimens prior to the event.	Moderate	Follow-up for the primary outcome was reasonably complete (mean 43.3 months); some ancillary serologic testing was incomplete but this does not affect the primary diagnosis.	Low	Outcomes ascertained via explicit validated criteria (2017 McDonald, Miller causality criteria, certified EDSS scoring) - a rigorous, objective approach.	Low	All identified eligible cases are presented, with only one explicit, reasoned a priori exclusion.	Serious
Kalén et al. [31], 2022	Single-arm retrospective referral cohort (Sweden)	D1: Serious | D2: Serious | D3: Low | D4: Moderate | D5: Low | D6: Low | D7: Low	Serious	No unexposed comparator group exists and the authors themselves state epidemiological risk estimation is not possible from this design; softened from Critical because the study still meaningfully improves outcome-ascertainment accuracy for the review (see D6).	Serious	Patients entered only via referral for suspected TNFi-associated neurological symptoms, an outcome-dependent sampling frame inherent to a referral-based case-finding design.	Low	TNFi exposure was verified through detailed medical-record review, with patients explicitly excluded when exposure could not be confirmed.	Moderate	No comparator arm exists and concomitant therapy at TNFi initiation is not detailed, though multi-agent exposure was transparently reported where present.	Low	Core exposure/outcome data were reasonably complete for the analyzed subgroup, with long mean follow-up (10-13 years).	Low	Outcome ascertained via objective 2017 McDonald criteria read by two independent evaluators; the study directly demonstrates and corrects for the unreliability of ICD-code-only ascertainment used elsewhere in this literature.	Low	Identified subgroups all appear reported without evidence of selective suppression.	Serious
